# Rational Design and Fabrication of ZnONPs Functionalized Sericin/PVA Antimicrobial Sponge

**DOI:** 10.3390/ijms20194796

**Published:** 2019-09-27

**Authors:** Lisha Ai, Huawei He, Peng Wang, Rui Cai, Gang Tao, Meirong Yang, Liying Liu, Hua Zuo, Ping Zhao, Yejing Wang

**Affiliations:** 1 Biological Science Research Center, Southwest University, Beibei, Chongqing 400715, China; als123@email.swu.edu.cn (L.A.); cairui0330@email.swu.edu.cn (R.C.); taogang@email.swu.edu.cn (G.T.); yangmeirong@email.swu.edu.cn (M.Y.); l3341345@email.swu.edu.cn (L.L.); zhaop@swu.edu.cn (P.Z.); 2 College of Biotechnology, Southwest University, Chongqing 400715, China; modelsums@email.swu.edu.cn; 3 Chongqing Key Laboratory of Sericultural Science, Chongqing Engineering and Technology Research Center for Novel Silk Materials, Southwest University, Beibei, Chongqing 400715, China; 4 College of Pharmaceutical Sciences, Southwest University, Chongqing 400715, China; zuohua@swu.edu.cn

**Keywords:** antimicrobial sponge, rational design, ZnONPs, cytocompatibility

## Abstract

The interests of developing antimicrobial biomaterials based on silk sericin from *Bombyx mori* cocoon, have been shooting up in the last decades. Sericin is a valuable natural protein owing to its hydrophilicity, biodegradability, and biocompatibility. Here, we fabricated a sponge with antibacterial capacities for potential wound dressing application. By co-blending of sericin, polyvinyl alcohol (PVA) and zinc oxide nanoparticles (ZnONPs), the ZnONPs-sericin/PVA composite sponge (ZnONPs-SP) was successfully prepared after freeze-drying. Scanning electron microscopy showed the porous structure of ZnONPs-SP. Energy dispersive spectroscopy indicated the existence of Zn in the sponge. X-ray diffractometry revealed the hexagonal wurtzite structure of ZnONPs. Fourier transform infrared spectroscopy showed the biologic coupling of ZnONPs and sericin resulted in a decrease of α-helix and random coil contents, and an increase of β-sheet structure in the sponge. The swelling experiment suggested ZnONPs-SP has high porosity, good hydrophilicity, and water absorption capability. The plate bacterial colony counting coupled with growth curve assays demonstrated that the composite sponge has an efficiently bacteriostatic effect against *Staphylococcus aureus* and *Escherichia coli*, respectively. Furthermore, the cell compatibility analysis suggested the composite sponge has excellent cytocompatibility on NIH3T3 cells. In all, ZnONPs-SP composite sponge has significant potentials in biomaterials such as wound dressing and tissue engineering.

## 1. Introduction

Antimicrobial sponge is a dramatically ascending research direction [1] since recent studies on nanotechnology manage to the synthesis of uniformly sized, well dispersed, and non-cytotoxic nanoparticles [2]. The unique physical and chemical properties grant the nanomaterials with many innate superiorities in biomedical applications [3]. Biological coupling of nanomaterials with sponge endows the carriers a splendid antibacterial property [4].

Sericin is a crude macromolecular protein from silk cocoons produced by the silkworm, *Bombyx mori* [5]. The role of sericin is to bond silk elements together to form a fibrous binary structure [6,7]. In the traditional silk reeling process, the soluble sericin is discharged into wastewater. About 50,000 tons sericin is wasted each year all over the world [8], causing water eutrophication and severe environmental pollution. As a natural protein, sericin is rich in various amino acids [9,10,11]. The predominant amino acid is serine (~30%), which makes it hydrophilic [12]. Sericin is of great value for its multiple properties such as anti-oxidation, UV absorption, and moisture retention properties. In addition, it is biocompatible and biodegradable. Sericin is beneficial to cell adhesion and proliferation [13,14]. Sericin has shown great value in biomedical engineering [15,16]. Hence, it is necessary to develop comprehensive utilization of silk sericin to solve the problem of environmental pollution caused by sericin.

Mechanical wounds, chronic wounds, burns, and skin ulcers destroy the protective layer of the skin [17]. These wounds expose the skin directly to the environment, which makes it easily subject to bacterial infection if not treated in time. Wound healing begins almost immediately after skin damage occurs [18]. To avoid wound infection during the healing process, wound dressing is indispensable. An ideal wound dressing should have oxygen permeability, moisture retention property, and antibacterial properties to avoid wound secondary infection [19]. Also, it should be biocompatible. Sericin itself is difficult to form a certain shape for its amorphous structure [20]. To expand the applications of sericin, improving the toughness of sericin is required. Poly (vinyl alcohol) (PVA) is a promising non-toxic, hydrophilic and biocompatible polymer. PVA has strong mechanical properties, good thermal, and chemical stability [21,22]. After the addition of PVA, the mechanical properties of sericin are greatly improved [23].

Nanomaterials have broad application potential owing to its large surface area, high stability, catalytic and antimicrobial properties [24]. Zinc oxide (ZnO) is a safe compound admitted by the US Food and Drug Administration (21 CFR 182.8991). Zinc oxide nanoparticles (ZnONPs) have broad-spectrum antibacterial properties. ZnONPs will generate reactive oxygen species (ROS) under illumination. These active substances such as O^2−^, OH^−^, and H_2_O_2_ can result in fatal bacterial damage [25]. The antimicrobial activity of ZnONPs relies on the surface area to volume ratio. The smaller the particle size of ZnONPs (higher specific surface areas), the higher the antibacterial activity [26]. Another antibacterial mechanism of ZnONPs is the release of Zn^2+^ which could repress the active transport of membranes and destroy the enzyme systems and amino acid metabolism [27,28]. Hence, ZnONPs are widely applied in food packaging, cosmetics, and pharmaceutical industry [29].

In this paper, we combined the natural advantages of sericin, PVA and ZnONPs to fabricate antimicrobial sponges with great potentials in wound dressing. PVA was blended with sericin to form a composite sponge with a certain shape, which greatly makes up the disadvantage of sericin in mechanical performance. ZnONPs were integrated into the interior of sericin/PVA (SP) sponge as a slow-release antibacterial reagent. The prepared ZnONPs-SP sponge was characterized by scanning electron microscopy (SEM), energy dispersive spectroscopy (EDS), X-ray diffractometry (XRD), and fourier transform infrared spectroscopy (FTIR). The antibacterial properties of ZnONPs-SP were assessed against *E. coli* and *S. aureus*. The cytocompatibility of the composite sponge was evaluated by a mouse embryonic fibroblast cell line NIH3T3. Our results demonstrated that ZnONPs-SP sponge has a promising potential as a wound dressing for its excellent stability, antibacterial activity and good cytocompatibility.

## 2. Results

**Figure 1:**
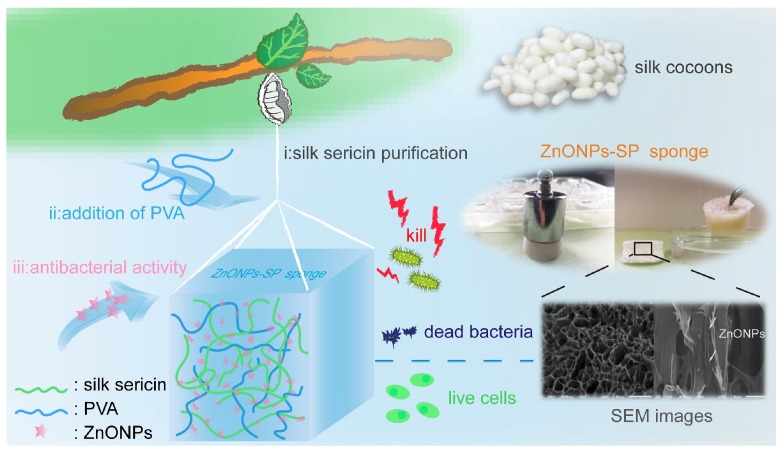
Illustration of the preparation and evaluation of ZnONPs-SP sponge.

In view of the natural advantage of sericin, we designed a green synthesis strategy to fabricate antibacterial ZnONPs-SP sponge for potential wound dressing application. Here, sericin and PVA were blended and crosslinked by hydrogen bond, which enhanced the toughness of sericin. Then, ZnONPs were absorbed into the sericin/PVA sponge by the effect of charge and biological coupling. The prepared ZnONPs-SP sponge has high porosity and swelling ratio, good antibacterial properties, and cytocompatibility, which has shown great potentials in the biomedical industry. The preparation and evaluation of ZnONPs-SP sponge were illustrated in Figure 1.

### 2.1. SEM, XRD, FTIR

**Figure 2:**
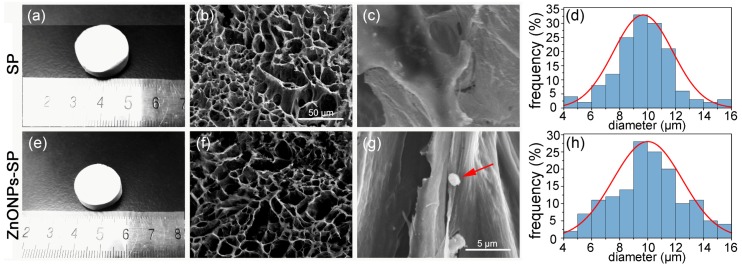
SEM. The pictures of SP sponge (**a**) and ZnONPs-SP sponge (**e**), SEM pictures of SP (**b**,**c**) and ZnONPs-SP (**f**,**g**). (**b**,**f**) cross-section, (**c**,**g**) longitudinal section, (**d**,**h**) the particle size analysis of SP and ZnONPs-SP, respectively. The red arrows indicated ZnONPs.

There was no difference in the appearance of SP sponge and ZnONPs-SP sponge (Figure 2a,e). SEM showed that there was no significant difference in the longitudinal and transverse sections between SP and ZnONPs-SP sponges (Figure 2b,c,f,g). The results showed that SP and ZnONPs-SP sponges had a relatively uniform pore size of 10 ± 6 μm (Figure 2d,h). The porous sponge could facilitate the exchange of air, provide enough space for cells growth and the release of active molecules [30]. The longitudinal section view of ZnONPs-SP showed the existence of ZnONPs, as indicated by the red arrow in Figure 2g. The results suggested the addition of ZnONPs did not affect the morphology and pore size of SP sponge. 

**Figure 3:**
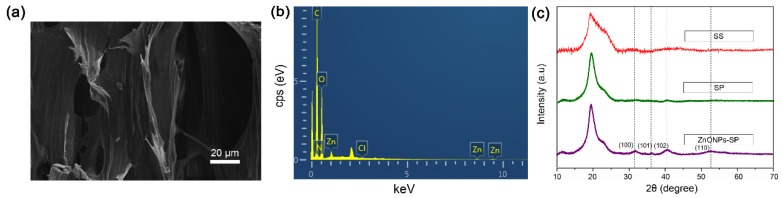
SEM, EDS and XRD. (**a**) Field emission scanning electron microscope image of ZnONPs-SP, (**b**) EDS spectrum of ZnONPs-SP, (**c**) XRD profiles of sericin, SP, and ZnONPs-SP sponge.

XRD pattern reflects the atomic and molecular structure of a crystal. EDS utilizes different X-ray photon feature energies of different elements for component analysis. To verify the elemental content of ZnONPs-SP sponge, we performed an EDS analysis. Figure 3a showed the morphology of a selected area in the ZnONPs-SP sponge. The EDS spectrum indicated the presence of Zn and O elements in the ZnONPs-SP sponge (Figure 3b). In addition, the XRD pattern showed distinct peaks at 2θ = 19.2°, 31.3°, 36.2°, 47.5°, and 56.6° (Figure 3c). The peak at 2θ = 19.2° is the featured peak of sericin [31,32]. The diffraction peaks observed at 2θ = 31.3°, 36.2°, 47.5°, and 56.6° correspond to the crystal planes (100), (101), (102), and (110) of ZnO, indicating the formation of hexagonal wurtzite structure in ZnONPs [33].

**Figure 4:**
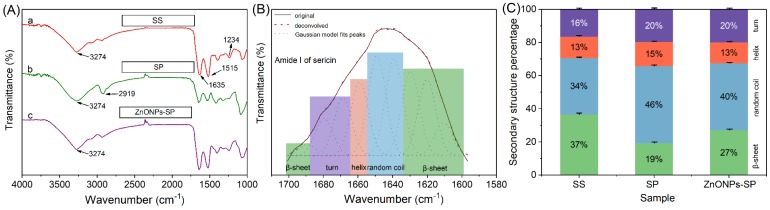
FTIR spectra and secondary structure analysis. (**A**) FTIR spectra of sericin (a), SP (b), ZnONPs-SP (c), (**B**) The curve-fitted spectrum of the amide I region of sericin, (**C**) The contents of different secondary structures in the sponges.

Figure 4A showed the FTIR spectra of sericin, SP and ZnONPs-SP sponges. The amide peak changes correspondingly with the secondary structure and molecular orientation of molecules. To study the effect of the composite sponge on the secondary structure of sericin, firstly, we analyzed the characteristic secondary structure of sericin. The wavenumber range of 1700–1600 cm^−1^ represents the characteristic peak I of sericin, which corresponds to C=O vibration [34]. In 1575–1480 cm^−1^, it is the characteristic peak II of sericin, corresponding to the vibration contraction of N-H [35]. Another typical peak of sericin is in 1301–1229 cm^−1^ [36]. The results showed that the peaks located at 1635 cm^−1^ (amide I), 1515 cm^−1^ (amide II), and 1234 cm^−1^ (amide III) were characteristic peaks of sericin (Figure 4A-a) [37]. The typical peak of the O-H stretching vibration is at 3274 cm^−1^ (Figure 4A) [38].

To investigate the effects of PVA and ZnONPs on the secondary structure of sericin, we further explored sericin characteristic amide I peak (1700–1600 cm^−1^) using Fourier auto-deconvolution and second-derivative algorithm [39]. Gaussian curves revealed sericin contains four typical secondary structures including random coil, *α*-helices, *β*-sheet, and *β*-turn (Figure 4B) [40]. The contents of various secondary structures in the sponges were shown in Figure 4C. *β*-sheet is the formation of parallel or antiparallel arrangements between two peptide chains or different parts of a peptide chain by means of hydrogen bonds. PVA could form hydrogen bonds with sericin so that it likely alters the hydrogen bonds between the amino acids of sericin. Hence, the content of *β*-sheet structure in SP reduced to 19% compared with that of sericin, while the content of the random coil increased to 46%. The biologic coupling between ZnONPs and sericin led to a decrease of *α*-helices and random coil proportion and an increase of *β*-sheet structure in the ZnONPs-SP sponge while compared to that in the SP sponge [41].

### 2.2. Porosity and Swelling Ratio

**Figure 5:**
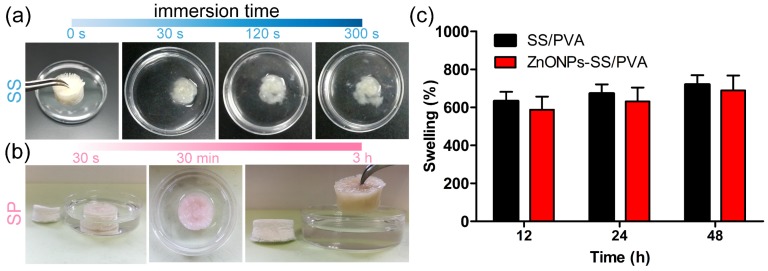
Porosity and swelling ratio. (**a**) The performance of sericin sponge in water, (**b**) The swelling of ZnONPs-SP sponge, (**c**) The swelling ratio of SP and ZnONPs-SP sponges.

As a potential wound dressing, the sponge should have the capability of retaining its natural shape after absorbing water. The morphology of sericin sponge was quickly changed in 300 s (Figure 5a), however, the physical appearance of sericin/PVA sponge was still intact even after 3 h (Figure 5b). The result implied the sericin/PVA composite had better performance than that of sericin. Porosity is one of the significant factors for cell growth and proliferation. The porosity of SP and ZnONPs-SP sponges were calculated to be 42% and 43%, respectively, suggesting ZnONPs does not affect the porosity of SP sponge.

The swelling ratio of SP and ZnONPs-SP sponges were 608% and 586% after 12 h, respectively. Increasing soaking time slightly improved the swelling ratio of the sponges (Figure 5c). There was no obvious difference in the swelling ratios between SP and ZnONPs-SP sponge, indicating ZnONPs does not affect the swelling ratio of SP sponge. In addition, the swelling ratio did not change over time, suggesting that the sponge was almost saturated in the first 12 h. Our results suggested ZnONPs-SP sponge had good hydrophilicity and water absorption performance.

### 2.3. Antibacterial Properties

**Figure 6:**
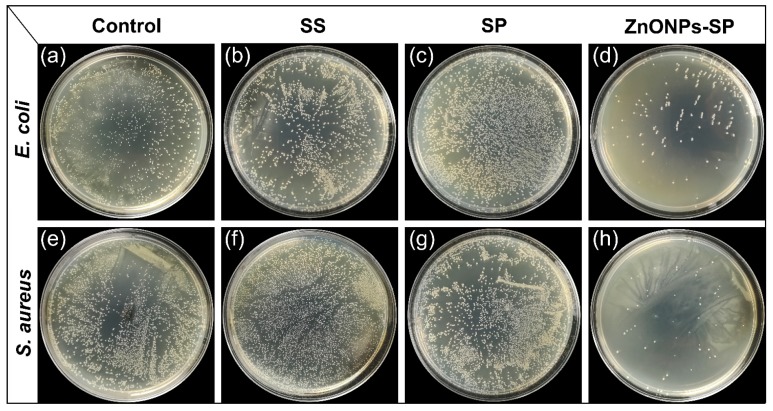
Bacterial colony counting assay. The antibacterial performance of control, sericin, SP, and ZnONPs-SP sponge against *E. coli* (**a**–**d**) and *S. aureus* (**e**–**h**). LB medium was used as the control.

An antibacterial sponge has broad application potential in the biomedical field. Here, *S. aureus* and *E. coli* were used to validate the antibacterial activity of ZnONPs-SP sponge. The number of bacterial colonies was used to evaluate the antibacterial effect of the composite sponge. There was no significant difference in the colony number among the groups of control, sericin, and SP. However, the number of colonies in the presence of ZnONPs-SP sponge was greatly reduced while compared to that on other plates (Figure 6), suggesting the addition of ZnONPs improved the antibacterial properties of SP sponge. This effect could be ascribed to that the nanoscale increased the contact area of ZnONPs with bacteria through the high surface area to volume, and easily acted on bacteria to achieve an antibacterial effect [42,43]. The reactive oxygen produced by ZnONPs also inhibited bacterial growth. In addition, zinc ion released from ZnONPs can penetrate bacterial cell membrane, thereby inhibiting the metabolic activity of bacteria.

**Figure 7:**
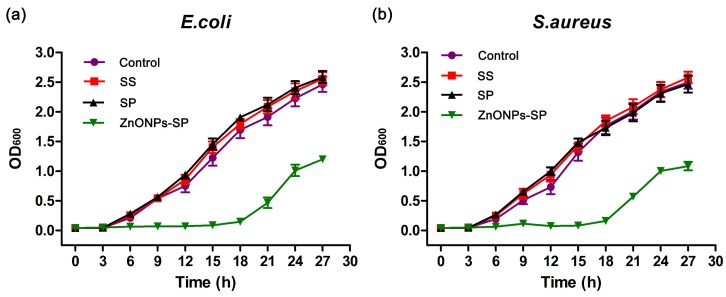
The growth curves of *E. coli* (**a**) and *S. aureus* (**b**) in the presence of the control, sericin, SP and ZnONPs-SP, respectively. LB medium was used as a negative control.

To further analyze the antibacterial effect of the composite sponge, we investigated the growth profile of *E. coli* and *S. aureus* in the presence of the sponges. The results showed that there was no significant difference in the bacterial growth among the control, sericin and SP sponge. However, the bacterial growth in the presence of ZnONPs-SP sponge was obviously inhibited while compared to other groups (Figure 7). The results of the bacteriostatic growth curves were consistent with that of the bacterial colony counting assay, indicating that ZnONPs-SP composite sponge has a prominent anti-microbial effect on *E. coli* and *S. aureus*.

### 2.4. Cytocompatibility

**Figure 8:**
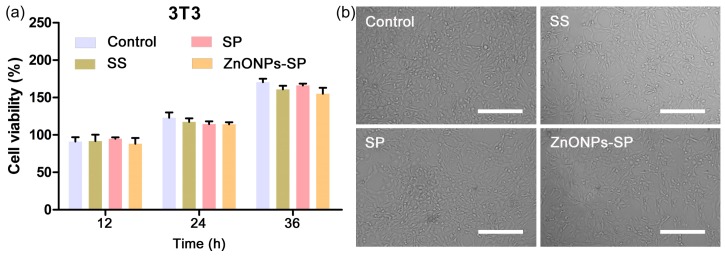
The cytocompatibility of ZnONPs-SP sponge. (**a**) CCK-8 analysis, (**b**) Cells morphology imaged on an optical microscope after 24 h. PBS was used as control. Scale bar, 200 μm.

The cytocompatibility of ZnONPs-SP composite sponge was valued after 24 h of incubation of the composite sponge with NIH3T3 cells at 25 °C. Figure 8a showed the viability of the cells did not have a significant difference among the control, sericin, SP, and ZnONPs-SP and control. Further, the cell images under optical microscope showed the cells had typical morphology including the appearance, distribution, shape and structure after 24 h of co-incubation with the control and the sponges (Figure 8b). The results suggested that ZnONPs-SP sponge has good cytocompatibility on the mouse embryonic fibroblast cell NIH3T3 and does not affect cells growth.

**Figure 9:**
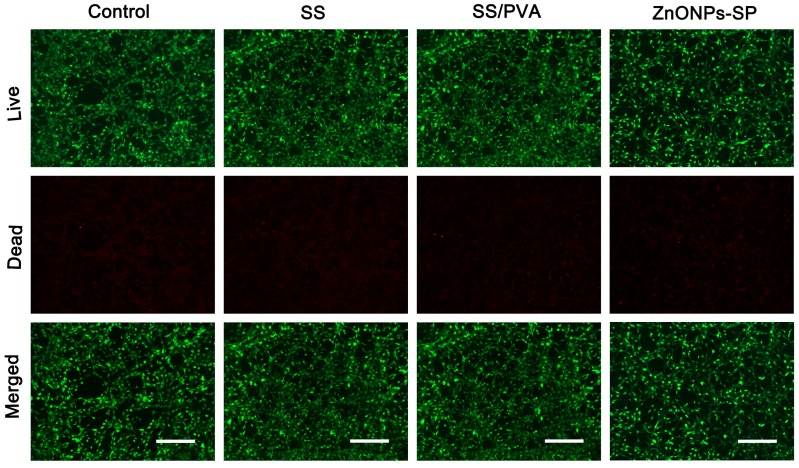
The LIVE/DEAD cells staining assay. PBS was used as control. Scale bar, 200 μm.

To better visualize the effect of ZnONPs-SP on cells viability, the LIVE and DEAD cells staining assay were carried out. The live and dead cells were dyed green and red, respectively. The merged images showed most of the cells were dyed green, only a few cells were dyed red (Figure 9), demonstrating the prepared ZnONPs-SP sponge had good cytocompatibility.

## 3. Discussion

Here, we fabricated an antibacterial ZnONPs-SP sponge derived from a natural macromolecule protein sericin. Sericin could easily mimic natural extracellular matrix [44], support cell attachment, promote cell proliferation [45,46] and has superior developmental competence of mammalian zygotes [47]. Hence, it can be readily used as a cell scaffold for wound dressing. Whereas, the natural sericin is brittle in nature. Therefore, the strategy of developing sericin based biomaterial is to design the co-polymeric system of artificial and natural polymers by blending or cross-linking with sericin [44,48]. Our previous study showed that chemical coupling could effectively improve the mechanical strength of sericin film by crosslinking with dialdehyde carboxymethyl cellulose [6]. However, the low toughness of the former fabricated sericin-material is still an enormous obstacle in front of further interests.

Sericin is usually combined with other materials, such as hyaluronic acid [49], sodium alginate [50], and chitosan [51] to improve its properties. In this way, the favorable properties of each polymer could be retained, meanwhile, the defects of each component could be remedied. In this study, sericin was blended with PVA to enhance the structural strength of the composite sponge due to the formation of the hydrogen bonds between sericin and PVA. ZnONPs have been widely applied in optical catalysis, energy, and other fields [26]. The bacteriostasis effect of ZnONPs is ascribed to the produced superoxide free radicals and the released Zn^2+^. In this work, the content of β-sheet in the ZnONPs-SP composite sponge increased to 27%, suggesting that the coupling of ZnONPs and sericin could induce the formation of β-sheet structure [41].

Here, we evaluated the biological effects of the antibacterial ZnONPs-SP sponge mainly from two aspects, antibacterial ability, and cell compatibility. The bacterial colony counting assay demonstrated the composite sponge has excellent antimicrobial activity, which is attributed to the good antimicrobial properties of ZnONPs [29]. The bacteriostatic growth curves showed that the compound sponges had similar inhibitory effects on both gram-negative and gram-positive bacteria [28]. In terms of cytocompatibility, cells viability tests confirmed that the viability of the cells in the presence of ZnONPs-SP sponge was not different from that in the presence of the control, sericin, and SP sponge, even after 48 h. Moreover, the Live/Dead cells staining assay showed clearly ZnONPs-SP sponge has excellent cytocompatibility on NIH3T3 cells.

In all, our results showed that sericin was well blend with PVA, and ZnONPs were successfully loaded into SP composite sponge. The bacteriostasis and cell compatibility tests demonstrated ZnONPs-SP composite sponge has good antimicrobial ability and excellent cytocompatibility. Our study greatly advanced the progress of sericin-based inorganic antimicrobial materials and the potential application in biomedical materials.

## 4. Materials and Methods

### 4.1. Materials

Silkworm cocoons were collected from the state key laboratory of silkworm genome biology, southwest university, Chongqing, China. ZnONPs were from Guohua reagent (Hansi, Shanghai, China). PVA was from Aladdin Corp (Shanghai, China). Cell counting kit-8 (CCK-8) and LIVE/DEAD cell viability kit were from Beyotime (Beijing, China). The reagents for cells culture including Dulbecco’s modified eagle’s medium (DMEM), trypsin-EDTA, fetal bovine serum (FBS) and penicillin/streptomycin were from Gibco (Waltham, MA, USA).

### 4.2. Preparation of ZnONPs-SP Sponge

Sericin was extracted into water from silkworm cocoons under 0.1 MPa at 120 °C for 20 min. Sericin solution was then lyophilized to become sericin sponge, and stored at −80 °C after the separation from fibroin by filtration. After that, PVA and sericin were dissolved in water at 80 °C with stirring to form a mixed solution. The concentration of sericin and PVA were 4% (*w*/*t*) and 5% (*w*/*t*), respectively. ZnONPs were dissolved in water and treated with ultrasonication for half an hour, then added into sericin/PVA (SP) solution. The final concentration of ZnONPs was 24.7 mM. The resultant ZnONPs/sericin/PVA solution was cast into a 24-well plate to form ZnONPs/sericin/PVA (ZnONPs-SP) sponge after freeze-drying.

### 4.3. Characterization of ZnONPs-SP Sponge

The surface morphology of ZnONPs-SP and SP were imaged on a Hitachi SU3500 (Tokyo, Japan). The accelerating voltage was 15 kV. The sponges were fixed on the aluminum stub using conductive liquid and sputter-coated with gold prior to tests [52].

The crystalline structure of the composite sponge has conducted on an X’ pert PRO X-ray diffractometer (Almelo, The Netherlands) over Bragg angles ranging from 10° to 80°.

The FTIR spectra of the samples were collected on a Thermo-Fisher Nicolet iz10 IR microscope (Waltham, MA, USA) in the region of 4000–400 cm^−1^. Every group was scanned for 16 times.

### 4.4. Porosity and Swelling Ratio

The porosity of SP and ZnONPs-SP sponge was measured by liquid displacement method [53]. The sponge was immersed in water in a 10 mL cylinder. The volume of water in the cylinder was recorded as *Vb*. The total volume of the sponge and water was recorded as *Vn*. After 10 min, the swollen sponge was taken out of water. The volume of the rest water was recorded as *Vs*. The difference between *Vb* and *Vs* indicated the volume of water absorbed by the sponge. The difference of *Vn* and *Vs* represented the volume of the swollen sponge. The test was repeated for three times. The porosity (*P*) of the sponge was calculated by the following equation:

*P* = (*Vb* − *Vs*)/(*Vn* − *Vs*) × 100% (1)

The swelling ratio of the sponge in phosphate buffer (PBS, pH 7.4) was measured as the gravimetric method [54]. The original sponge was weighed as *W*_0_. The sponge was immersed in PBS buffer at 37 °C. After 5 min, the sponge was taken out and gently wiped off excess water from the surface. The mass of the swollen sponge was defined as *Wa*. The experiment was repeated three times under the same condition. The swelling ratio of the composite sponge was the average of three experiments. The swelling ratio (*S*) was calculated by the following formula:

*S* = (*Wa* − *Wo*)/*Wo* × 100% (2)

### 4.5. Antibacterial Assay

The antibacterial performance of the sponge was carried out against *E. coli* and *S. aureus* [55]. The bacteria of experimental and control groups were cultured in the presence of the sponges at 37 °C. After 12 h, the bacteria were diluted 10 times with sterile water. Then the diluted bacteria were spread on a solid agar plate and cultured at 37 °C for 12 h. Finally, the antibacterial properties of the sponges were evaluated statistically according to the number of colonies on the plate.

The bacterial solution was diluted to an OD_600_ of 0.02. Then, the sterile SS, SP, and ZnONPs-SP sponges treated by UV light were added into the diluted bacterial solution. Then, the bacteria were cultured at 37 °C. The OD_600_ of the bacteria was measured every three hours to plot the growth curve of the bacteria. The assay was performed in triplicate for each independent experiment.

### 4.6. Cell Compatibility

NIH3T3 cells were cultured in DMEM medium supplied with 5% CO_2_ at 37 °C. The sponge was cut into small pieces and sterilized overnight with UV light. The cells were cultured in a 96-well plate in the presence of different sponges. To ensure the repeatability and accuracy of the tests, the initial cells density in each well was consistent. After 12, 24 and 36 h, NIH3T3 cells were measured for the viability with cell counting kit-8 (CCK-8), respectively. The CCK-8 reagent was added into each well to incubate with the cells at 37 °C as the manufacturer’s instruction. After 1.5 h, the optical density (OD) of each well was measured at 450 nm on a Glomax Multi Detection System (Mannedorf, Switzerland). The cells viability of each group was calculated from three independent tests. Further, the cytotoxicity of the sponge on NIH3T3 cells was evaluated by LIVE & DEAD assay. After 24 h, the cells were incubated with live/dead cell staining reagent at 37 °C for 2 h. Then, the cells were observed on an Olympus fluorescence microscope IX71 (Tokyo, Japan). Each experiment was repeated at least three times independently.

## 5. Conclusions

In summary, we fabricated an antibacterial ZnONPs-SP composite sponge by rational design. The design well combined the advantages of sericin, PVA and ZnONPs. The prepared ZnONPs-SP sponge has high porosity, good hydrophilicity and water absorption ability. In addition, the composite sponge has exhibited excellent antibacterial properties and cytocompatibility. These properties will benefit the development and utilization of sericin based biomaterials.
